# Biomarkers in Islet Cell Transplantation for Type 1 Diabetes

**DOI:** 10.1007/s11892-018-1059-4

**Published:** 2018-09-05

**Authors:** Fatimah T. AlRashidi, Kathleen M. Gillespie

**Affiliations:** Diabetes and Metabolism, Bristol Medical School, University of Bristol, Level 2, Learning and Research, Southmead Hospital, Bristol, BS10 5NB UK

**Keywords:** Biomarker, Type 1 diabetes, Cell-free DNA, microRNA 375, Islet transplantation

## Abstract

**Purpose of Review:**

Islet transplantation, an important approach to achieve insulin independence for individuals with type 1 diabetes, is limited by the lack of accurate biomarkers to track beta-cell death post islet infusion. In this review, we will discuss existing and recently described biomarkers.

**Recent Findings:**

As beta cells are killed by the immune system, fragments of beta cell-specific cell-free DNA and proteins are released into the periphery. Several different strategies to identify these fragments have been described. Some circulating, non-coding microRNAs, particularly miRNA-375 are also showing potential to reflect the rate of beta cell loss post-clinical islet transplantation.

**Summary:**

Recent advances in identifying accurate beta cell-specific biomarkers such as differentially methylated insulin cell-free DNA and circulating miRNA-375 may help predict clinical outcomes. More studies are required to examine the robustness of these biomarkers to detect chronic beta-cell loss in islet transplantation recipients.

## Introduction

Type 1 diabetes (T1D) is a chronic autoimmune disease that results from specific immune-mediated destruction of the insulin-producing beta cells in the pancreatic islets of Langerhans. Uncontrolled hyperglycemia characterises this autoimmune disorder, due to insufficient insulin production. Although not the most common form of diabetes (accounting for approximately 10% of all diabetes), T1D is the most common form of diabetes among children and adolescents under the age of 20 [1]. According to the most recent report from the International Diabetes Federation (IDF) in 2017, the incidence rate of T1D is increasing by 3% annually with approximately 132,600 new cases registered each year. There are about 1.1 million children and adolescents under the age of 20 years living with the condition globally, half of whom live either in Europe (28.4%) or North American/Caribbean regions (21.5%). Prediction studies from EURODIAB-registered children revealed that the incidence of T1D is projected to double in European children diagnosed under the age of 5 years between the years 2005 and 2020, and T1D prevalence will increase up to 70% in children under age 15 years [2]. Additionally, analysis of time trends by the Nationwide Diabetes Incidence Study in Sweden (DISS) [3] showed that the incidence of T1D onset in individuals aged 0–34 years from 1983 to 2007 is shifting to younger ages [4]. Taken together, these data suggest a growing population of young people with T1D. Clinical symptoms such as thirst, frequent urination and fatigue [1] appear when approximately 70–80% of an individual’s beta cells have been destroyed [5, 6]. At this point, the only option for the patient is lifelong insulin replacement and blood glucose monitoring. Insulin replacement does not work equally for all recipients. Exogenous insulin is not as exquisite as native insulin in controlling blood sugar levels, and the Diabetes Control and Complications Trial (DCCT) showed that there is an association between glucose homeostasis and the risk of developing chronic T1D micro and macrovascular complications [7, 8] highlighting the importance of good blood glucose control.

## The Pancreatic Beta Cell

The beta cell is a hormone-secreting cell that forms a significant component of the islets of Langerhans in the pancreas (approximately 60–80%) [9]. In addition to beta cells, the islets contain four types of endocrine hormone-producing cells: 30% alpha cells (secreting glucagon), < 10% delta cells (secreting somatostatin), < 5% gamma cells (secreting pancreatic polypeptide) and epsilon cells (secreting ghrelin) [9–13]. The islets compose the endocrine pancreas and account for only 1–2% of the total mass of the human pancreas [9]. Anatomical and three-dimensional (3D) visualisation showed that the endocrine pancreas is uniquely heterogeneous with regard to islet number and size. The total number of islets range between 3.6 and 14.8 million, and their volume varies from 0.5 to 1.3 cm^3^ [9, 14–16]. The total number of islets is highest in the body/tail of the pancreas [9].

## T1D Reversal Strategies

Currently, the primary approach to attempt to cure T1D is by restoring beta-cell mass and controlling the ongoing autoimmune reaction. To date, there are two clinically available methods for restoring beta cell mass; the first is whole pancreas transplantation; the second is through transplantation of only the islets of Langerhans, both of which are usually harvested from brainstem death donors. These methods of beta-cell replacement are therefore only offered to patients with ongoing difficulties in controlling their blood glucose levels through insulin management [17]. According to the international pancreas transplant registry, the overall survival rates among their Pancreas After Islet (PAI) transplantation recipients (*n* = 40) were 97%, 1- year post-transplantation, and 83%, 5-year post-transplantation [18]. The registry reported that the current patient survival rate at 3-year post-transplantation is more than 93% with graft function in the simultaneous pancreas and kidney recipient greater than 83% and solitary pancreas graft > 70% [19•]. Nevertheless, the process involves major surgery, prolonged hospitalisation and the need for life-long immunosuppressive therapy [18].

## Enhanced Islet Transplantation Protocol–Edmonton Protocol

Unlike whole pancreas transplantation, islet transplantation is a non-invasive strategy to treat T1D, as it does not require major surgery or prolonged hospitalisation; the patient will, however, require ongoing immunosuppressive medication. A multicentre, randomised controlled trial by the TRIMECO study group compared the efficiency of islet transplantation to the intensive insulin treatment in patients with severe hypoglycaemia or hypoglycaemia unawareness. The results showed that 84% (21 of 25) of islet transplantation recipients had restored normoglycemic levels 6-month post-islet transplantation, while 0% of patients who received intensive insulin attained such enhancement. The trial also showed that 59% of islet graft recipients (27 of 46) achieved insulin independence 1-year post-transplantation [20].

Clinical islet transplantation is primarily performed by infusing self or donated islets into the patient’s portal vein from where the islets are distributed throughout the liver. An innovative immunosuppressive treatment and cautious preparation of islets allowed the researchers at the University of Alberta (Edmonton, Canada) to achieve complete insulin dependence for seven patients with T1D, 1-year post-islet transplantation [21]. The “Edmonton Protocol” used an immunosuppressive combination of glucocorticoid-free drugs comprising sirolimus, tacrolimus and daclizumab as well as enhanced methods of islet isolation, with an emphasis on transplanting a sufficient number of islets for the recipient. The recommended islet mass for infusion is 5–7000 islet equivalents (IEQ)/kg of recipient’s body weight from two or more donated pancreases [22, 23]. The Phase 3 Trial of the Clinical Islet Transplantation Consortium (CIT-07) had registered 48 T1D patients (T1D duration > 5 years) in eight islet transplantation centres who underwent islet engraftment. Their report showed that 87.5% of the registered patients achieved normal glucose levels (HbA_1c_ > 7%) 1-year post the first islet infusion and 71% remained normoglycemic two years later [24•, 25].

According to the Collaborative Islet Transplant Registry, more than 1500 patients have received islet transplantation worldwide [22]. Advances in donor selection, islet isolation and enhanced immunosuppression regimes have resulted in long-term insulin independence in more than 50% of clinical islet transplantation recipients mirroring the outcomes of the whole-organ pancreas transplantation in selected international centres [22, 26]. It is also worth highlighting here that more studies are required to optimise immunosuppression strategies as the majority of adverse events associated with the islet transplantation procedure originated from immunosuppression medications [20].

## Monitoring Graft Outcomes

Despite the encouraging outcomes from islet transplantation on enhancing the quality of life of patients with T1D who previously had difficulties controlling blood glucose levels, in general, however, islet function decreases over time in islet transplant recipients and repeat transplants are required. The procedure can be negatively impacted by significant cell loss during and immediately after islet infusion due to instant blood-mediated inflammatory responses, cell metabolic exhaustion and hypoxia [27–29]. Long-term, the clinical outcome is hampered by a recurrence of islet autoimmunity and rejection. Efforts to improve the outcome include strategies to increase islet engraftment and to protect transplanted islets from the host immune system. Biomarkers are crucial to monitor ongoing graft function, but no validated direct measure exists to reliably monitor the rate of engrafted cell death, and this is a limitation of the procedure.

Currently, clinical islet transplantation relies on the metabolic biomarkers such as the levels of blood glucose, haemoglobin A1C (HbA1C), stimulated C-peptide and daily insulin measurement to assess the function of the graft [30]. Although it is not affected by exogenous insulin, C-peptide measurement has several limitations as an indicator of transplant rejection; it is influenced by factors that have impact insulin secretion, such as insulin resistance [31]. Further, it is well established that C-peptide often fails to reflect the presence of beta-cell destruction during the earlier pre-diabetic stage and therefore is only useful for gross changes in beta-cell function. The ability to measure subtle changes in the rate of beta-cell death in islet transplant recipients would inform clinical interventions.

Autoantibodies to the islet antigens insulin (IAA), glutamic acid decarboxylase (GADA), islet cell antigen-2 (IA-2A) and zinc transporter 8 (ZnT8A) are unsurpassed as biomarkers to predict future T1D but their use as biomarkers in islet transplantation has been less definitive. Increases in autoantibody level with epitope spreading were associated with an adverse outcome [32], but this has not been reported by all centres, so long-term follow-up with harmonised islet autoantibody assays is required to establish the predictive capacity of islet autoantibody level and characteristics in the islet transplantation setting.

Alloimmunity is monitored through measurement of donor-specific autoantibodies (DSA) against donor HLA antigens but results from studies to date have proved confusing. DSA positivity before transplantation has been associated with graft failure [33, 34], but not by all studies [32, 35] while the study of the appearance of DSA post-transplant has also proven controversial [32, 33, 36–39]. As with islet autoantibody analysis, further collaborative studies are required to understand the true potential usefulness of DSA in islet transplantation. The lack of current real-time biomarkers to measure the efficacy of preventative and interventional therapeutic approaches still poses an immense challenge in islet transplantation. Recently, evidence has accumulated showing that biomarkers such as microRNA and differentially methylated cell-free DNA of key beta cell genes have the potential to be powerful tools to evaluate transplantation success and graft function.

A biomarker is an indicator that objectively measures a normal or pathological state as well as evaluating biological responses to a therapeutic intervention [40]. Ideally, a biomarker should have some specific criteria to be of clinical value; it should be with highly selective and specific to a target, stable, unaffected by physiological or metabolic changes, have a detectable threshold in the circulation and be reproducible. Above all, a biomarker should be non-invasive, acceptable to the patient and easily interpreted by the clinician (Table 1) [41]. Furthermore, an acceptable biomarker should apply to different populations, have a series of cut-off values in a general population and be detectable in the early stage of the disease. Extra considerations should be weighed up for diagnostic and therapeutic biomarkers: the expense, the difficulty of sample collection and the patient disturbance during sample collection. For example, a biomarker destined to be used in routine screening tests should have the advantage of both easy access from patients and minimum cost per sample. On the other hand, diagnostic and research study biomarkers should be highly specific for the target specimen such as a tissue biopsy. In that case, the number of samples processed will be limited, but the specificity and the sensitivity will be at their maximum [41].

**Table 1:** Features and characterisation of the ideal biomarker

Characterisation	Comments
Specific	Have the ability to differentiate pathological from normal state
	Specific to the affected cells or tissue
Sensitive	Rapid and Significant release upon disease development
Anticipative	Long half-life in biological sample
	Its release is proportional to the degree disease severity
Robust	Rapid, simple, accurate and inexpensive detection
	Not changed by environmental factors and other diseases
Non-invasive	Present in easy accessible biological fluid samples
	Minimum risk to the patient at the time of sample collection

## Circulating Proteins in Assessing the Outcomes of Clinical Islet Transplantation

Glutamic acid decarboxylase 65 (GAD65) was one of the first candidate biomarkers to be suggested for detection of beta cell death in models of diabetes [42, 43]. A preclinical report studying the level of variant biomarkers for monitoring beta cell death post auto- and allo-islet graft into dogs had reported that enzymatic assay for GAD achieved limited success in predicting of islet graft rejection; nevertheless, the authors were optimistic about the possibility of detection of islet-specific proteins during preclinical stage of the diabetes [44]. Human GAD65 is reported to be expressed in neuroendocrine cell types, brain and pancreatic alpha, beta and delta cells [42]. In patients receiving an intraportal islet allotransplant elevated GAD65 was detected within hours of surgery, but more sensitive assays are required to detect more subtle episodes of cell death that may inform graft rejection or recurrent diabetes [45].

The development of more sensitive detection methods, such as time-resolved fluorescent immunoassay technologies (TRFIA) [46] have shown some promise for predicting the outcomes of clinical islet transplantation, but it was insufficient to consistently detect GAD65 in all recipients [45, 47]. More recently, development of advanced immunoassay platforms such as the Cytometric Bead Array (CBA), the ElectroChemiLuminescence ImmunoAssay (ECLIA) and the digital ELISA technology (Single Molecule Array-SIMOA) had improved the sensitivity of the assay for detecting circulating GAD65 in the sup-picomolar range [48]. More studies are required, however, to test the utility of these advanced technologies in measuring beta cell death and predicting future outcomes in islet transplantation recipients. In addition, one concern is that the use of GAD65 as a biomarker might be limited by the presence of circulating GAD65 autoantibodies in the periphery of T1D islet recipients.

## MicroRNA in Assessing the Outcomes of Clinical Islet Transplantation

MicroRNAs have multiple distinctive features, and providing that they are genuinely tissue-specific, they could be potentially powerful biomarkers for evaluating changes in health and disease. MicroRNAs are abundant in multiple easy-to-collect biological samples such as urine, saliva, tears, seminal fluid and breast milk [49]. Moreover, previous studies showed that microRNA levels are amenable to measurement using different methodologies [50–55]. Accumulating data emphasise a role for circulating microRNAs (miR) in controlling the physiological pathways of insulin secretion and beta-cell survival [56].

miR-375 was one of the first identified, most abundant islet-specific microRNAs and also one of the best-characterised microRNAs regarding its function [57]. *NeuroD1/BETA2* and *PDX-1* both synergistically control the expression of miR-375 [58]. miR-375 plays a fundamental role in normal glucose homeostasis, alpha- and beta-cell turnover, and adaptive beta-cell expansion in response to increasing insulin demand in insulin resistance [57, 59, 60]. Measuring the level of miR-375 to monitor beta cell death has been described previously [61–63] when the absolute and relative levels of miRNA-375 were associated with the level of beta cell damage in vivo and in vitro [64]. Piemonti and colleagues had previously suggested that circulating miR-375 levels could potentially be a non-invasive biomarker for monitoring beta cell death post-islet transplantation. A total of 22 human islet infusions were studied under different immunosuppression treatments. They reported that serum miR-375 had upregulated 200-fold within the first 12 h of infusion, and then decreased to baseline level 24 h post-infusion. A second peak was reported at 96 h preceded by elevated C-reactive protein and cross-linked fibrin degradation products, and followed by an increase in the cell damage markers aspartate aminotransferase, alanine aminotransferase and lactate dehydrogenase [65]. Subsequently, the Naziruddin group also studied the level of miR-375 in patients undergoing pancreatectomy with islet autologous or allogeneic transplantation (*n* = 29). They showed that miR-375 was significantly elevated within 3 h post-infusion. Additionally, they demonstrated that the rising level of miR-375 mirrored the rising level of C-peptide post-islet infusion [64]. Later, the same group was able to confirm their previous conclusion showing that miR-375 was a reliable indicator for predicting islet death post-transplantation. Following up a total pancreatectomy with islet autotransplantation (TPIAT) patients for 1-year post-transplantation (*n* = 31) revealed that miR-200c correlates with the islet metabolic transplantation outcomes. The data showed that high relative miR-200c expression (< 1.5) was associated with a favourable engraftment function and with less insulin dependence [66]. These early observations need to be followed up with large longitudinal studies.

## DNA Methylation in Assessing the Outcomes of Clinical Islet Transplantation

**Fig. 1:**
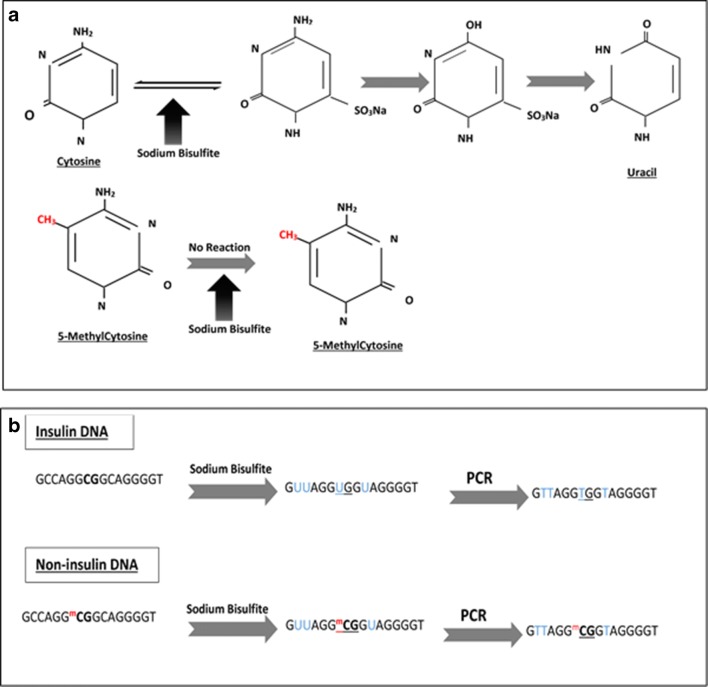
Sodium bisulfite conversion of genomic DNA. This allows methylated and unmethylated residues to be discriminated. **a** The conversion occurs on denatured, single-stranded DNA with sodium bisulfite at high temperature and low pH (pH = 5). The reaction starts when the unmethylated 6th carbon atom of the cytosine is sulfonated, then, irreversible hydrolytic deamination occurs at the 4th carbon atom that converts the molecule into uracil sulfonate, and, finally, a succeeding desulfonation occurs in an alkaline environment converting the molecule into a uracil nucleotide. The cytosine methylation at 5th carbon atom obstructs the first sulfonation step of the bisulfite methylation reaction protecting the cytosine nucleotide from conversion. **b** The sodium bisulfite chemical reaction converts the unmethylated cytosines* at position − 69 (from the Transcription Start Site) of insulin gene into uracil which is replaced by a thymine nucleotide during the PCR reaction, whereas methylated cytosines in other non-insulin DNA sequence remain as cytosine. *The methylation occurs in CpG dinucleotide (bold) in the human genome

Differentially methylated DNA-based biomarkers of beta cell-specific genes have been reported to correlate with the rate of beta-cell death in recently diagnosed T1D, those who are at risk for developing the disorder and assessing the outcomes of novel clinical therapies [67–71, 72••, 73, 74••]. Dying beta cells during the pathology of T1D release their DNA content into the circulation. There are some specific cytosines within this DNA that have a differential methylation pattern that allows the cell of origin of the DNA to be identified. These unique methylation signatures are mainly within the genes that are vital for cell function and identity maintenance. As shown in Fig. 1, well-established methodologies exist to identify the differentially methylated sites. To date, studies that employed differentially methylated DNA-based biomarkers to assess the outcomes of islet transplantation have focused on the beta-cell-specific insulin gene (*INS*) (Table 2). Husseiny and colleagues in 2014 followed up islet transplant patients (*n* = 6) and showed that beta cell-specific hypomethylated *INS* cell-free DNA (cfDNA) was significantly increased a day after transplantation; according to their data, the elevated level persists for at least 14 days post the infusion [70]. Differentially hypomethylated CpG sites within the *IN*S promoter were also used to interpret the outcomes of allogeneic islet transplantation in longstanding T1D patients by Lehmann-Werman and colleagues [72••]. They showed that beta cell-specific cfDNA was detected in the plasma of islet recipients 1–2 h post-infusion; the level declined sharply in the following hours and days in a manner that was in parallel with the result seen previously in imaging studies on islet transplantation recipients [76]. More recently, another group using the same approach used by Lehmann-Werman observed a correlation between beta cell-specific cfDNA levels post-clinical islet allograft at a specific time point and the graft outcomes [75••]. They reported that there were two waves of high beta cell-specific cfDNA signals in their islet graft recipients (*n* = 37); the first was a temporary and a highly intense signal seen immediately post-infusion (within 1 h). This instant signal is thought to be caused by cell hypoxia and the innate inflammatory responses. The second elevated signal is less intense and more informative, observed 24 h post the infusion. This latter signal was suggested by the authors to be a predictor of islet engraft rejection as a higher level of hypomethylated *INS* signal was linked to unfavourable graft outcomes [75••]. The level of beta cell-specific *INS* cfDNA was also studied in patients undergoing TPIAT. A study by Herold and colleagues [69, 74••] showed that the relative level of hypomethylated *INS *cfDNA was significantly raised in the first 3 h post-islet infusion in TPIAT patients. Variable late beta cell-specific signals were observed among the recipients, as well over the following 30 days post the infusion suggesting inconsistency in graft loss [74••]. They also reported that 30% of recipients showed a high signal on day 90 post the infusion, which was suggested by the authors to be associated with unfavourable graft outcomes. The use of cfDNA as biomarkers (increasingly advanced in the cancer field) are in their infancy in diabetes research. Improved standardised beta cell-specific assays are required to truly define their future clinical usefulness.

**Table 2:** Potential methylated DNA biomarkers for islet engraftment

	Cohort number, sample type	Human data				Reference
		Patient age (years)	Tested time range	Technique	Conclusion	
Circulating proteins						
GAD65	(*n* = 26) Plasma	N/A	0–24 h	Time-resolved fluorescence immunoassay	1. Plasma GAD65 was detected in the first 24 h post infusion in proportion to the dying beta cell mass during the graft preparation procedure. 2. Plasma GAD65 was not detected in all graft recepients as the assay had limited sensitvity (functional sensitivity of 0.174 ng/mL)	[45]
microRNA expression						
miR-375	(*n* = 22) serum	N/A	N/A	N/A	1. Serum miR-375 increased 200-fold within the first 12 h of infusion 2. A second peak was observed 96 h post the infusion that paralleld upregulation in C-peptide level, cross-linked fibrin degradation products and cell damage markers	[65]
miR-375	(*n* = 29) Sera	Autologous: 36.6–41.6 Allogeneic: 43.2–52.4	1–3–6-7-24 h 3–5–7 days	SYBR Green quantitative PCR	In combination with C-peptide proinsulin level, miR-375 is a potential robust biomarker that monitors beta cell death in clinical islet transplantation.	[64]
miR-375 and miR-200c	(*n* = 31) Plasma	31.9–51.7	1 year post infusion	SYBR Green quantitative PCR	miR-375 is a potentially promising biomarker for predicting islet damage. miR-200c could be a novel biomarker for predicting metabolic graft function post the TPIAT procedure.	[66]
Unmethylated *INS* DNA^a^						
CpG -206 & CpG-135	Plasma and whole Blood samples	41–53	Day 1 and day 14	Nested methylation-specific PCR	Whole blood samples: The level of unmethylated *INS* rose significantly 1-day post the graft; the signal remained elevated 14 days post the graft. Plasma samples: The level of unmethylated INS was significantly elevated 1-day post the graft, and fell by the 14th day after the graft.	[70]
6 CpG sites within the *INS* promoter	(*n* = 10) Plasma	44–57	1–24 h to 7 days- 1 month	Illumina MiSeq Sequencing	A significant sharp signal was observed 1-2 h post islet transplantation. Signals above the background level detected in some patients seven days and one month post-transplant may indicate ongoing beta cell loss.	[72••]
CpG +396 & CpG +399	(*n* = 21) Serum	17.6–44.6	15–30–60 min 3–6–12–24 h 3–5–7–14–21–30–90 days	Droplet Digital PCR	Unmethylated INS assay is a potentially powerful marker that detects the rate of islet damage following clinical islet transplantation.	[69, 74••]
6 CpG sites within the *INS* promoter	(*n* = 37) Plasma	26–63	1 to 24 h to 7 days to 1 month	Illumina MiSeq Sequencing	1. Two waves of signals were observed First: strong and transient at 1-h post graft Second: less intense and less frequent but predictive for the future engraftment rejection at 24 h post graft 2. Higher unmethylated signals at the 24-h time point was positively associated with a larger islet packed cell volume (PCV).	[75••]

^a^The CpG position is identified in relation to human assembly genome GRCh37/hg19; Feb 2009

## Conclusion

Islet transplantation has been shown to be a promising therapeutic approach that enhances the quality of life of patients with T1D. The lack of accurate and efficient biomarkers to monitor beta-cell death and predict engraftment function and transplantation rejection still poses an immense challenge. In recent years, biomarkers such as circulating miRNA-375 and differentially methylated insulin cfDNA have emerged as potentially powerful tools to detect real-time beta cell injury post islet transplantation. A significant advantage of these biomarkers is that they provide a real-time rate of beta-cell death during and immediately after the islet infusion. Unlike metabolic biomarkers, differentially methylated biomarkers and microRNAs level are not affected by external physiological changes or internal metabolic alteration. Nonetheless, the identification of a panel of accurate, reliable and robust beta cell-specific biomarkers is still in its infancy. Full validation of these biomarkers is required in clinical islet transplantation trials.
